# Multifunctional Biosensing Platform Based on Nickel-Modified Laser-Induced Graphene

**DOI:** 10.3390/bioengineering10050620

**Published:** 2023-05-21

**Authors:** Yao Tong, Yingying Zhang, Benkun Bao, Xuhui Hu, Jiuqiang Li, Han Wu, Kerong Yang, Senhao Zhang, Hongbo Yang, Kai Guo

**Affiliations:** 1 School of Biomedical Engineering (Suzhou), Division of Life Sciences and Medicine, University of Science and Technology of China, Hefei 230026, China; 2 Suzhou Institute of Biomedical Engineering and Technology, Chinese Academy of Sciences, Suzhou 215163, China

**Keywords:** flexible electronics, non-invasive monitoring, multi-biomedical signal sensor, laser-induced graphene

## Abstract

Nickel plating electrolytes prepared by using a simple salt solution can achieve nickel plating on laser-induced graphene (LIG) electrodes, which greatly enhances the electrical conductivity, electrochemical properties, wear resistance, and corrosion resistance of LIG. This makes the LIG–Ni electrodes well suited for electrophysiological, strain, and electrochemical sensing applications. The investigation of the mechanical properties of the LIG–Ni sensor and the monitoring of pulse, respiration, and swallowing confirmed that the sensor can sense insignificant deformations to relatively large conformal strains of skin. Modulation of the nickel-plating process of LIG–Ni, followed by chemical modification, may allow for the introduction of glucose redox catalyst Ni_2_Fe(CN)_6_ with interestingly strong catalytic effects, which gives LIG–Ni impressive glucose-sensing properties. Additionally, the chemical modification of LIG–Ni for pH and Na^+^ monitoring also confirmed its strong electrochemical monitoring potential, which demonstrates application prospects in the development of multiple electrochemical sensors for sweat parameters. A more uniform LIG–Ni multi-physiological sensor preparation process provides a prerequisite for the construction of an integrated multi-physiological sensor system. The sensor was validated to have continuous monitoring performance, and its preparation process is expected to form a system for non-invasive physiological parameter signal monitoring, thus contributing to motion monitoring, disease prevention, and disease diagnosis.

## 1. Introduction

Flexible wearable physiological signal monitoring sensors have a wide range of applications in many medical and health fields, such as daily physiological monitoring, disease prevention, and disease diagnosis and treatment [1,2,3,4,5,6]. The design of physiological parameter monitoring equipment needs to take into account the diversity of physiological parameters, the high demand for continuous real-time detection of physiological signals in medical treatment and prevention, as well as the comfort of physiological parameter detection. Flexible wearable testing devices have great demand and development momentum because of their non-invasive monitoring and multi-parameter continuous real-time monitoring capability [7,8,9]. The main physiological parameters monitored by non-invasive flexible sensing are commonly divided into three components: electrochemistry (e.g., sweat pH, sweat ion concentration), electrophysiology (e.g., electromyogram (EMG), electrocardiogram (ECG), electroencephalogram (EEG)), and mechanical strain (e.g., pulse, respiration, vocalization) [10,11]. Electrochemical sensing mainly monitors sweat, usually using materials with high surface electrochemical properties and specific chemical modifications to achieve rapid extraction of effective sweat information [12]. Electrophysiological signals are now commercially monitored mainly with wet electrodes, which can achieve a high signal-to-noise ratio (SNR) and good repeatability [13,14]. Recent studies have shown that non-invasive, high signal-to-noise ratio, and high temporal resolution bioelectric signal monitoring can be achieved by optical monitoring [15,16]. Mechanical strain is mainly monitored with highly sensitive piezoelectric sensing and tensile sensors [17,18].

Considering the effect of the miniaturization of multiple physiological parameters monitoring sensors [19], a more uniform sensor substrate preparation scheme can minimize the integration difficulty of physiological sensors and reduce the mutual interference caused by the difference between various physiological sensor preparation methods [13]. Laser-induced graphene (LIG) is a conductive material with a fast and controllable preparation process. As a material with a great specific surface area (SSA), good electrical conductivity, and certain tensile strain properties, LIG has been applied in three major non-invasive physiological monitoring directions and is currently an important material for the preparation of integrated sensors for multi-parameter monitoring [4,14,20]. The LIG electrode, as a typical dry electrode, has stronger stability compared with conventional the Ag/AgCl wet electrode because it is not affected by gel volatilization. A small amount of silver ions will be released during the use of the Ag/AgCl wet electrode, which will easily cause skin allergy and inflammation reactions. LIG dry electrodes do not contain common allergens, so they have a better safety profile than traditional wet electrodes [14].

There is a large gap between the conductivity of LIG sensors and conventional metal conductors. However, the monitoring of physiological signals is carried out through sensors that convert various physiological parameters into electrical parameters. So, the conductivity of the sensors will directly affect the quality of the signals. To improve the conductivity of LIG electrodes while retaining the high specific surface area properties, a more feasible method is to enhance the conductivity of LIG through conductive modification. Nickel is a metal material that is widely found in nature that has the high electrical conductivity common to metals and the ability to passivate. The surface of nickel plating generated by electroplating produces a very thin passivation film that resists the corrosive effects of air and acid. Meanwhile, nickel plating is simple and inexpensive. A great crystalline layer can be obtained by plating with a simple salt electrolyte. The high hardness and wear resistance of the nickel crystalline layer also allow the electrodes modified by nickel plating to work stably on the skin. Therefore, the conductive modification of LIG electrodes by electroplating nickel can achieve enhanced electrode conductivity, corrosion resistance, and wear resistance [21]. During nickel plating, Ni(OH)_2_ deposition on LIG electrodes can be formed by adjusting the concentration of the electrolyte composition. Ni(OH)_2_ has been shown to catalyze the process of glucose conversion to gluconolactone under alkaline conditions [22]. The simultaneous modification of Prussian blue by electrochemical methods may allow for the conversion of Ni(OH)_2_ to Ni_2_Fe(CN)_6_ on the sensor. Shi-Kui Geng et al. showed that Ni_2_Fe(CN)_6_ is a high-performance urea oxidation electrocatalyst. The composites containing Ni_2_Fe(CN)_6_ also have good electrocatalytic properties for glucose [23]. Therefore, using the nickel-plating method can enhance the conductivity, stability, and electrochemical functions of LIG materials, which offers the possibility of preparing high-performance multi-physiological parameter monitoring systems at a low cost by using a more uniform preparation process.

Results and discussions will present the mechanical properties of the LIG–nickel-plated (LIG–Ni) sensor starting with the laboratory preparation method. The preparation process and monitoring the performance of the sensors for different physiological parameters are also described. Sensing using the LIG–Ni sensor is demonstrated from three application perspectives: electrophysiological, strain, and electrochemical in this part. The conclusions section will systematically summarize the advantages of the preparation and application of LIG–Ni sensors in the field of physiological signal monitoring, and look forward to their application.

## 2. Results and Discussion

### 2.1. Preparation of LIG–Ni Flexible Sensor Manufacturing

**Figure 1:**
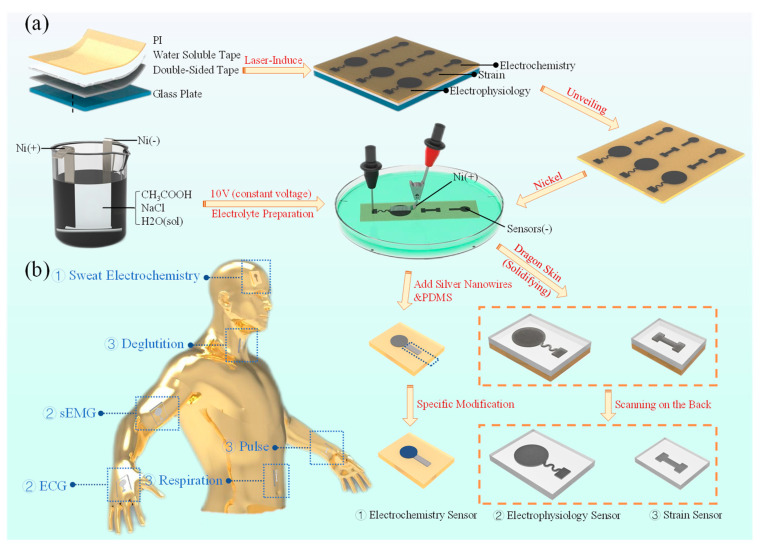
Preparation and application of three LIG–Ni sensors. (**a**) Schematic diagram of the complete preparation process of the three sensors (including paste plate preparation, laser-induced graphene, electrolyte preparation, Ni plating, and subsequent preparation process of different functional sensors). (**b**) Schematic diagram of the application of three sensors for non-invasive monitoring (the serial number ①②③ corresponds to electrochemistry sensor, electrophysiology sensor and strain sensor).

The preparation part of the LIG–Ni sensor fabrication is divided into two basic steps: (i) plate making and LIG generation, (ii) electrolyte preparation and Ni plating. After completing the nickel plating on the LIG surface, the sensor can be modified according to different physiological parameters to be measured. LIG preparation was accomplished by using PI films to generate graphene through the laser-induced process. The polyimide (PI) film is kept in the laser focus plane during the LIG preparation by attaching it to a flat plate. This makes the LIG preparation process easier to manipulate and results in a more homogeneous LIG material. Usually, a four-layer structure of PI, water-soluble tape, double-sided tape, and glass plate are used for overlapping and attaching the board. Laser-induced formation of a thin layer of graphene fibers on the surface of PI films is observed. The use of dry preserved water-soluble tape allows the PI layer to be easily peeled off from the lower substrate by wetting with a small amount of water after the LIG has been generated (Figure 1a).

The process of electroplating Ni on the prepared LIG substrate requires the preparation of electrolytes. The electrolyte is made of a mixture of CH_3_COOH (1 M, 3 g/50 mL) and NaCl (0.34 M, 1 g/50 mL), and is electrolyzed with a constant voltage source of 5 V DC under the condition that both the cathode and anode are Ni. During the electrolyte preparation process, the Ni sheet located at the anode will gradually dissolve to form Ni^2+^ into the solution, while fine H_2_ bubbles are generated by the cathode. As the electrolysis proceeds, the electrolyte gradually changes to light lime green, which is evidence of the generation of Ni^2+^ within the solution during electrolysis. The main ionic equations in the preparation of the electrolyte are as follows:

Anode: Ni − 2 e^−^ → Ni^2+^

Cathode: 2 H^+^ + 2 e^−^ → H_2_(g)

The Ni modification on the LIG surface can be completed by connecting the Ni electrode (anode) and the LIG electrode (cathode) in the prepared electrolyte and plating with a 10 V DC power supply. Sensor substrates that have completed the Ni modification process are cleaned using deionized water. After drying, it can be chemically modified to have the ability to monitor electrochemical parameters, such as pH, glucose concentration, and ion concentration levels of sweat. It is also possible to prepare flexible sensors (e.g., electrophysiological sensors, strain sensors) by coating flexible materials (e.g., Dragon Skin) for LIG–Ni layer immobilization and then removing the PI by laser scanning on the back. The sensor takes advantage of the high conductivity of the Ni modification to obtain higher signal quality of physiological signals.

### 2.2. Electromechanical Characterization

**Figure 2:**
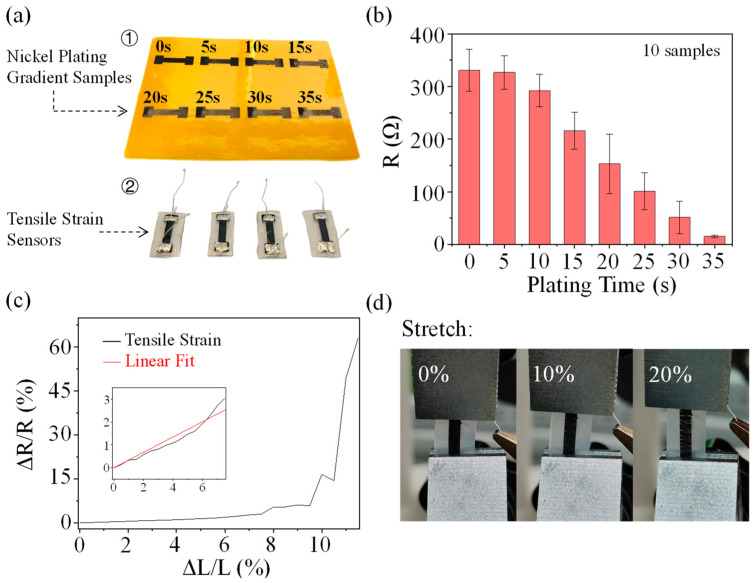
Mechanical characteristics of the LIG–Ni sensor. (**a**) LIG–Ni mechanical property test samples (including bone rod pattern LIG–Ni test samples with ①different nickel-plating times and ②the same shape tensile strain sensors). (**b**) The mean standard deviation chart of resistance-plating time for ten LIG–Ni samples. (**c**) Line chart of the rate of change of resistance with tensile strain for saturated nickel-plated LIG–Ni sensors. (**d**) Uniform cracks on the surface of saturated nickel-plated sensors after tensile rates greater than 10%.

To investigate the effect of nickel plating on the electrical conductivity of LIG, a bone rod pattern LIG was prepared for nickel plating gradient experiments (Figure 2a). The experiments were carried out using a controlled time scheme to control nickel plating amount, and the gradient experiments were increased by 5 s per gradient. The resistance of the middle part of the bone rod model obtained from several experiments was all saturated when the nickel-plating time reached 35 s. In the saturated nickel-plated condition, the resistance of this bone rod model is about 15.2 Ω.

Data from 10 collected samples are illustrated in the mean standard deviation chart, where the maximum and minimum data points at each time have been removed. Resistance detection sites are located at both ends of the bone rod midline. It can be seen that as the amount of nickel plating increases, the conductivity of LIG also goes up, which positively promotes the nickel-plating process to proceed. The peak rate was reached at about 20 s of nickel plating. Additionally, the LIG–Ni electrode resistance gradually stabilized as the amount of nickel plating became saturated. The unplated samples’ surface resistance was 3304 (Ω/cm^2^) on average, and the surface resistance of the samples in the saturated nickel-plated state was 151.5 (Ω/cm^2^) on average, which is about 20 times lower relative to the unplated ones. This proves that the nickel-plating process greatly improves the electrical conductivity of LIG.

LIG can withstand 20% strain and maintain electrical conductivity, and its conductivity curve with tensile strain usually has a J-shaped distribution. When stretched to a certain length, LIG conductivity decreases sharply with further stretching changes, which is mainly caused by the generation of cracks during the stretching process [24]. Tensile tests were performed after saturated nickel plating of LIG (Figure 2c). The Ni coating leads to a more stable tensile strain around 10% strain for LIG–Ni (Figure 2c), which narrows the corresponding range of LIG. Therefore, Ni plating can cause LIG–Ni embrittlement. Fine cracks appear in the flexible bone rod structure at a 10% stretching state, and uniform stretching cracks appear when increasing the stretching to 20%, making the sensor close to being non-conductive (Figure 2d). So, the conductivity and tensile strain performance of LIG–Ni can be regulated by controlling the amount of nickel plating. The LIG–Ni material with a larger amount of nickel plating is more suitable for the design of flexible conductive structures for the region with less strain, while the LIG–Ni material with a smaller amount of nickel plating is more suitable to be used in the region with a larger strain range. The LIG–Ni obtained with saturated Ni plating retains a certain strain-insensitive range, and the design of stretchable structures such as serpentine can enhance the resistance stability of saturated Ni-plated LIG–Ni during stretching and increase the stretch rate threshold. LIG–Ni can be used selectively in flexible conductors or strain sensors by controlling the amount of nickel plating in consideration of strain range and conductivity requirements.

### 2.3. LIG–Ni Application for Electrophysiological Monitoring

**Figure 3:**
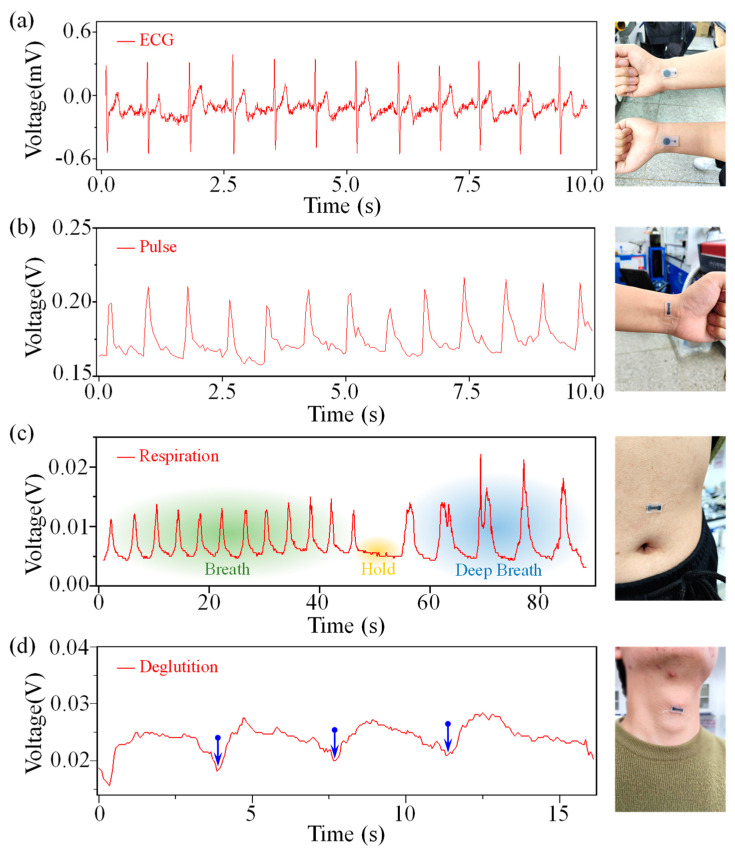
LIG–Ni sensor for real-time electrophysiological and tensile strain parameter monitoring. (**a**) Sampling data for real-time monitoring of ECG signals on the inner wrist (left). Schematic of the location of the electrophysiological sensor attachment (right). The left part of (**b**–**d**) shows samples of real-time strain monitoring signals for a pulse, abdominal breathing, and swallowing. The right part shows the corresponding monitoring sites for three-strain detection.

Transferring LIG–Ni using flexible and highly biocompatible materials can prepare electrophysiological sensors to validate their application in electrophysiology and strain parameter monitoring. The design of the LIG–Ni sensor for electrophysiology is shown in the right part of Figure 3a. The sensor consists of a serpentine conductive part with a circular sensor body. The LIG–Ni electrophysiological sensor can be attached to the inside of the wrist or the back of the hand for ECG monitoring. If the electrolyte for nickel plating is kept for a long time, the acetic acid and water in the solution will evaporate, causing the solution acidity to drop and the Ni^2+^ concentration to increase, which will lead to the formation of Ni(OH)_2_ crystals on the surface of LIG during the plating process. Since this sensor needs to be in contact with skin, and nickel salts are commonly carcinogenic, the amount of nickel released from the LIG–Ni sensor needs to be controlled [25]. So, it is necessary to strictly ensure that the electrolyte is freshly made. The PI layer can be removed together with the sensor part by dissolving the water-soluble tape under the PI layer. After LIG–Ni is covered by the highly biocompatible flexible material, the LIG can be detached from the PI film by laser scanning on the back to complete the basic preparation of the electrophysiological sensor.

Normally, the electrophysiological signal monitoring sites are not subject to large deformations. LIG–Ni sensors prepared using a saturated nickel-plating method can maximize the performance of dry electrode electrophysiological parameter monitoring by improving conductivity. Compared with the Ag/AgCl wet electrode, this LIG–Ni dry electrode is less irritating to the skin. Additionally, the use of flexible and thin substrate transfer allows it to retain a better conformability with skin, which also ensures its great monitoring capability. The left part of Figure 3a shows the continuous ECG signal obtained by using the LIG–Ni electrophysiological sensor processed with low-frequency band-pass filtering and power frequency filtering. The PQRST wave of the ECG can be easily recognized from the chart, proving that the sensor can be effectively used for ECG monitoring [26].

### 2.4. LIG–Ni Application for Strain Monitoring

The bone rod-shaped LIG–Ni flexible strain sensor, prepared using a preparation process similar to that of the electrophysiological sensor, can be used to monitor strain physiological parameters such as pulse, abdominal breathing, and swallowing [27,28]. Since the LIG–Ni strain sensor requires a constant current source, a layer of RT GEL 4317 flexible material can be applied to the LIG–Ni side of the sensor to improve the conformal adhesion of the sensor to the skin while avoiding direct stimulation by electric current. The LIG–Ni strain sensor can obtain stable and high-quality pulse wave signals through continuous monitoring by inner wrist attachment, which confirms the capability of the LIG–Ni sensor for minimal deformation monitoring (Figure 3b). The attachment position of this LIG–Ni strain sensor applied to abdominal respiratory monitoring is shown in the right part of Figure 3c. Meanwhile, obvious signal frequency and threshold changes have been found in the conversion between three phases, so three respiratory patterns could be effectively distinguished by monitoring information. This indicates that it has the potential to monitor the breathing status and even to warn against apnea (Figure 3c). In addition, the signal acquisition process of this LIG–Ni strain sensor applied to swallowing is shown in the right part of Figure 3d. The sensor is applied laterally to the neck to monitor sudden changes in the sensor signal during swallowing, which validates that swallowing monitoring can be achieved by setting a threshold value adapted to the individual. This holds the promise of real-time, effective swallowing monitoring for many patients with chronic diseases that can cause swallowing disorders, such as stroke and Parkinson’s [28].

### 2.5. LIG–Ni Application for Electrochemical Monitoring

In the electrolyte solution generated by the electrolysis of nickel using an acetic acid and sodium chloride solution, the H^+^ concentration in the solution decreases due to the electron gain of H^+^ at the cathode to H_2_(g). If the electrolyte is kept for a longer time, the volatility of acetic acid will further cause the solution to be less acidic. The evaporation of water from the electrolyte also leads to an increase in Ni^2+^ concentration. During nickel plating using an electrolyte prepared for two days, the use of a higher plating voltage causes additional electrolytic water processes, which result in the enrichment of hydroxyl radicals at the cathode. Under the effect of this series of alkaline enhancing influences, a longer time of nickel plating will lead to Ni(OH)_2_ deposition on the cathode LIG due to the low solubility product of nickel hydroxide (Ksp(Ni(OH)_2_): 2.0 × 10^−15^). Plating Prussian blue using LIG–Ni electrode with Ni(OH)_2_ deposition aids to resolve this issue. Since the OH^−^ concentration of the Prussian blue plating solution is not low enough and the solubility product of Fe(OH)_3_ is much smaller than that of Ni(OH)_2_ (Ksp(Fe(OH)_3_): 3.2 × 10^−38^), the Ni(OH)_2_ is converted into Fe(OH)_3_ in the process of plating Prussian blue. The chemical equation of the reaction is presumed to be:

Fe(CN)_6_^4−^ + 2 Ni(OH)_2_ + 4 H^+^ → Ni_2_Fe(CN)_6_ + 4 H_2_O

Ni_2_Fe(CN)_6_ is a refractory compound and can be used for the electrocatalysis of glucose redox in sweat. In addition, loading on a porous LIG–Ni surface can maximize its catalytic performance and speed up the electrochemical response [22,29,30]. It has been shown that Prussian blue has good electrochemical reversibility with high stability and good applications in electrocatalysis. The introduction of Prussian blue can further stimulate the glucose redox catalytic performance on the electrode surface.

**Figure 4:**
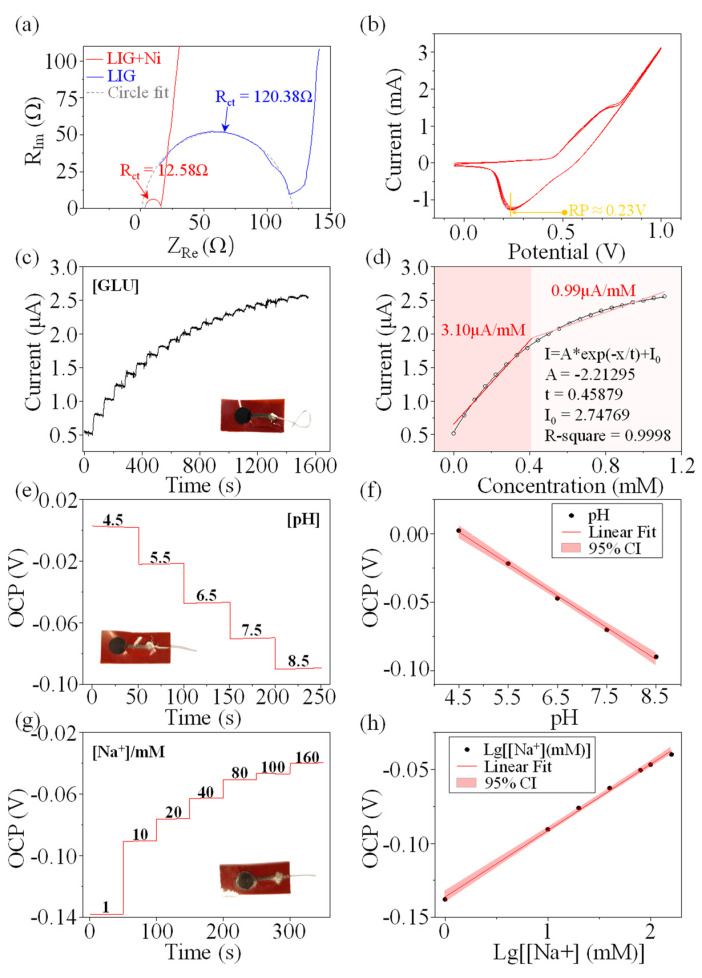
LIG–Ni sensor for electrochemical monitoring. (**a**) The electrochemical impedance spectroscopy (EIS) of LIG and LIG–Ni confirms that the charge transfer resistance (R_ct_) of LIG was nearly 10 times that of LIG–Ni. (**b**) Cyclic voltammetry (CV) of LIG–Ni scans in NaOH (0.1 M) solution. The reduction potential is about 0.23 V. (**c**) Glucose gradient sensing detection using a LIG–Ni enzyme-free glucose sensor. (**d**) Glucose concentration gradient detection results: piecewise linear fitting and exponential (ExpDec1) fitting. (**e**,**g**) The gradient detection of pH and concentration of Na^+^. Respectively, (**f**,**h**) are linear fitting of pH and Na^+^ concentration gradient detection results.

The charge transfer resistance (R_ct_) of the LIG electrode without nickel plating is nearly ten times higher than that of the electrode after nickel plating, which proves that LIG–Ni has much stronger electrochemical properties than LIG (Figure 4a). The LIG–Ni glucose sensor is prepared by rinsing the prepared nickel-plated electrode with deionized water, and then plating with Prussian blue by using constant voltage. Usually, the catalytic decomposition process of enzyme-free glucose takes place under alkaline conditions [31,32,33]. Cyclic voltammetry (CV) scans using 0.1 M NaOH as a solvent were obtained in Figure 4b. The electrode reduction potential was about 0.23 V. A constant pressure of 0.2 V was applied and glucose concentration monitoring was performed in the same NaOH concentration solution with a glucose gradient of 0.05 mM (Figure 4c). Glucose was added in single drops at an interval of 75 s, and then 10 s of steady current before each drop was taken to be the statistic obtained after the previous drop. The average value within 10 s was taken to make a dot plot, and the fit to the point plot was performed using ExpDec1 and a linear fit was performed in two intervals to obtain Figure 4d. Observe that the data trend is monotonically increasing, and the increasing rate is decreasing, so ExpDec1 is taken for fitting. In the high sensitivity region (glucose concentration range: 0 mM~0.37 mM) the linear fit glucose detection sensitivity was 3.60 μA/mM with an R^2^ of 0.9801; in the lower sensitivity region (glucose concentration range: 0.37 mM~1 mM) the glucose detection sensitivity was 1.07 μA/mM with an R^2^ of 0.9672. The exponential fitting curve is obtained as shown in Figure 4d, where R^2^ is 0.9998. When testing with this electrochemical sensor, segmented linear fitting or exponential fitting results can be selected according to specific accuracy requirements. The glucose concentration in sweat (usually below 1 mM) has been shown to correlate with that of blood. Therefore, the LIG–Ni electrochemical sensor can be well suited for sweat glucose detection studies and has good application prospects for non-invasive glucose monitoring [12,34].

The pH sensor can be prepared by constant pressure plating using a LIG–Ni electrode in an aniline–hydrochloric acid mixture solution. The detection results are shown in Figure 4e, and the linear fitting results are shown in Figure 4f. The pH value has a strong linear relationship with the open circuit potential. Therefore, the pH sensor prepared by using the LIG–Ni electrode can perform pH monitoring of sweat. The pH value of sweat is in the range of 4.5~5.5, which is within the linear monitoring range of the electrode. Monitoring of sweat pH can be used to assist in monitoring human health conditions, such as for characterizing dehydration and skin conditions [35].

The Na^+^ concentration sensor was prepared by using a LIG–Ni electrode in a PEDOT: PSS conductive polymer for constant pressure plating and then covering the electrode surface with a Na^+^ selective permeation film. The data of Na^+^ concentration monitoring experiments are recorded in Figure 4g, and the linear fitting results of the monitoring results are shown in Figure 4h [36]. The linear relationship between log concentration and open-circuit voltage was excellent in the range of 0~160 mM, which completely covered the floating range of Na^+^ concentration in sweat (10 mM~70 mM) [37].

This shows that LIG–Ni electrodes prepared using the nickel-plating method are extremely promising for non-invasive sweat monitoring of sweat glucose, pH, and common electrolytes. It has been investigated that sweat can be collected cyclically by sweat collection devices [8,38,39,40,41]. It is foreseen that equipping the collection device with electrochemical sensors prepared using LIG–Ni with appropriate electrochemical modifications can enable multi-parameter monitoring of sweat, thus promoting the development of non-invasive physiological monitoring in the electrochemical direction.

## 3. Conclusions

The electrical properties of sensors prepared using LIG–Ni sensors increase rapidly with the amount of nickel plating. A higher amount of nickel plating is suitable for the preparation of components with better conductivity while a lower amount can be used for the preparation of sensors with a larger strain range. The LIG–Ni electrode is advanced in the following five aspects: (i) through the nickel plating process, the thin oxide layer formed on the nickel surface can improve the corrosion and wear resistance of the LIG–Ni sensor, thus increasing the sensor life of servicing; (ii) the nickel plating improves the electrical conductivity of the LIG–Ni sensor, which makes it more adaptable to the monitoring of various physiological parameters; (iii) through chemical modification, Ni_2_Fe(CN)_6_ may be introduced on the surface of the LIG–Ni sensor as a strong catalyst, thus improving monitoring response of LIG–Ni sensors as an enzyme-free glucose sensor; (iv) the relatively uniform preparation process of electrophysiological, strain, and electrochemical LIG–Ni sensors also makes it easier to integrate multi-physiological parameter sensing systems; (v) the low-cost and simple fabrication process also make it easier to mass-produce LIG–Ni sensors. The LIG–Ni sensor has multiple excellent sensing properties, and its low-cost and easy preparation process can greatly improve the convenience of manufacturing non-invasive physiological parameters monitoring sensors. It is expected that it will have an impressive application in the field of non-invasive monitoring in the future.

## 4. Methods

### 4.1. Board and LIG Preparation

First, attach a glass plate (10 cm × 10 cm) with double-sided adhesive, then attach water-soluble adhesive, and finally attach PI film (thickness: 50 μm) to complete the production of the board.

The prepared board PI layer was processed upwards by laser processing equipment (universal Laser VLS3.50 Laser System) for LIG patterning, with the processing parameters: grating mode; power: 8%; rate: 15%; PPI: 1000.

### 4.2. Electrolyte Preparation

Take deionized water: 50 mL, pure acetic acid: 2.857 mL (1 M), and NaCl: 1 g (0.5 M) and mix well to obtain the mixed solution. Take two Ni sheets (about 0.2 g) and insert them into both ends of the solution. Use a constant voltage of 5 V connected to both ends of the nickel sheet and react for about 20 min (negative bubble generation can be observed). The positive nickel sheet consumes 0.08 g.

### 4.3. LIG Plated Ni Layer

Pour the prepared electrolyte into the plating cell, with the Ni sheet as the positive electrode and the LIG to be plated as the negative electrode, and immerse both electrodes in the solution. Apply a voltage of 10 V to both electrodes and sweep the Ni sheet across the LIG surface repeatedly at a uniform speed at a distance of about 1 mm near the LIG. After finishing Ni plating, rinse the LIG–Ni surface well with deionized water.

### 4.4. Fabrication of LIG–Ni Electrophysiological Sensor

Take 3.5 g of Dragon Skin 30 A and 3.5 g of Dragon Skin 30 B, mix thoroughly, and lay the mixture flat on the surface of the LIG–Ni sensor board. Use the screed machine to shake the board at 150 r/s for 30 s, and then leave it to cure for 2–3 h.

After curing, use a pipette gun to wet the water-soluble tape using deionized water, and remove the PI layer. Scan the PI layer on the back using the laser (universal Laser VLS3.50). Processing parameters: grating mode; power: 22%; rate: 100%; PPI: 1000.

After scanning, remove the PI layer and transfer the silver nanowire homogenate to the conductor portion by applying a capillary glass tube. Cut 2 cm of silver wire and paste it onto the end of the sensor using conductive silver paste. Use a heating table to heat the conductive silver paste at 90 °C until it dries. Take 0.1 g of hardener and 1 g of polydimethylsiloxane (PDMS) and mix thoroughly to obtain mixed glue. Use tweezers to take an appropriate amount of mixed glue to cover the conductive silver paste modification area and the front of the wire, and let it stand for 1 h to cure. The LIG–Ni electrophysiological sensor was fabricated by cutting the sensor into squares.

### 4.5. Fabrication of LIG–Ni Strain Sensors

The Dragon Skin transfer process and the laser scanning on the back process are the same as the corresponding fabrication process for the LIG–Ni electrophysiological sensor. Cut about 2 cm of silver wire and paste it on the end of the sensor using conductive silver paste, and then heat it at 90 °C using a heating table until the conductive silver paste is dry. Then, take 1 g of RT GEL 4317 A and 1 g of RT GEL 4317 B and mix well. Use tweezers to take an appropriate amount and apply it thinly and evenly on the side surface of LIG–Ni, and let it stand for 1 h to cure. Then, the LIG–Ni strain sensor is cut to a square to complete the fabrication.

### 4.6. Fabrication of LIG–Ni Electrochemical Sensor

The conductor part of the sensor is coated with silver nanowire homogenization, and the end is soldered with silver wire.

#### 4.6.1. Fabrication of LIG–Ni Glucose Sensor

The Ni plating step requires the electrolyte to be left for 48 h to allow the acetic acid to evaporate properly before plating.

Preparation of Prussian blue plating solution: DI water: 50 mL; 36% HCl: 0.45 mL (0.1 M); FeCl_3_: 0.02 g (2.5 mM); K_3_[Fe(CN)_6_]: 0.04 g (2.5 mM); KCl: 0.37 g (0.1 M) [42]. After configuring the Prussian blue plating solution, connect the Ag/AgCl reference electrode, counter electrode, and sensor (working electrode) to the electrochemical workstation, and submerge the three electrodes in the Prussian blue plating solution. Open the electrochemical workstation together with the chi660e software (Shenzhen, China) and set to the constant voltage deposition mode (0.6 V, 40 s).

#### 4.6.2. Fabrication of LIG–Ni pH Sensor

Configure a mixture of 0.1 M aniline and 0.1 M HCl. Similarly connect the three electrodes and submerge all three electrodes into the mixed solution. Set the chi660e software to the constant pressure deposition mode (0.6 V, 150 s).

#### 4.6.3. Fabrication of LIG–Ni Na^+^ Sensor

Configure the PEDOT: PSS (0.1 M EDOT with 0.1 M NaPSS). Similarly connect the three electrodes and then set the chi660e software to the constant current deposition mode (0.2 mA, 2800 s). Then add 2 μL of the Na^+^ selective transmission reagent dropwise to the sensor surface and dry naturally.

## Data Availability

The data presented in this study are available on request from the corresponding author.
